# Inhibition of tumour necrosis factor alpha by Etanercept attenuates Shiga toxin-induced brain pathology

**DOI:** 10.1186/s12974-025-03356-z

**Published:** 2025-02-07

**Authors:** Robin Christ, Devon Siemes, Shuo Zhao, Lars Widera, Philippa Spangenberg, Julia Lill, Stephanie Thiebes, Jenny Bottek, Lars Borgards, Andreia G. Pinho, Nuno A. Silva, Susana Monteiro, Selina K. Jorch, Matthias Gunzer, Bente Siebels, Hannah Voss, Hartmut Schlüter, Olga Shevchuk, Jianxu Chen, Daniel R. Engel

**Affiliations:** 1 Institute for Experimental Immunology and Imaging, University Hospital Essen, Essen, Germany; 2 Leibniz-Institut Für Analytische Wissenschaften, ISAS, E.V., Dortmund, Germany; 3 Life and Health Sciences Research Institute (ICVS),, School of Medicine, University of Minho, Braga, Portugal; 4 ICVS/3B’s, PT Government Associate Laboratory, Braga/Guimarães, Portugal; 5 Section Mass Spectrometry and Proteomics, Diagnostic Center, University Medical Center Hamburg-Eppendorf, 20246 Hamburg, Germany; 6 Institute of Molecular Medicine and Experimental Immunology, University Hospital Bonn, Bonn, Germany

## Abstract

Infection with enterohemorrhagic *E. coli* (EHEC) causes severe changes in the brain leading to angiopathy, encephalopathy and microglial activation. In this study, we investigated the role of tumour necrosis factor alpha (TNF-α) for microglial activation and brain pathology using a preclinical mouse model of EHEC infection. LC–MS/MS proteomics of mice injected with a combination of Shiga toxin (Stx) and lipopolysaccharide (LPS) revealed extensive alterations of the brain proteome, in particular enrichment of pathways involved in complement activation and coagulation cascades. Inhibition of TNF-α by the drug Etanercept strongly mitigated these changes, particularly within the complement pathway, suggesting TNF-α-dependent vasodilation and endothelial injury. Analysis of microglial populations using a novel human-in-the-loop deep learning algorithm for the segmentation of microscopic imaging data indicated specific morphological changes, which were reduced to healthy condition after inhibition of TNF-α. Moreover, the Stx/LPS-mediated angiopathy was significantly attenuated by inhibition of TNF-α. Overall, our findings elucidate the critical role of TNF-α in EHEC-induced brain pathology and highlight a potential therapeutic target for mitigating neuroinflammation, microglial activation and injury associated with EHEC infection.

**Graphical Abstract:**

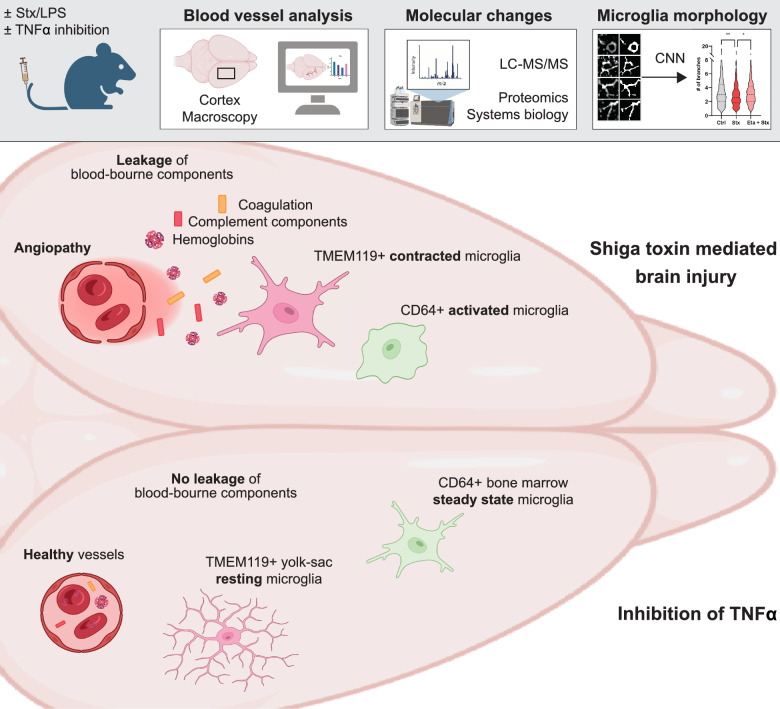

**Supplementary Information:**

The online version contains supplementary material available at 10.1186/s12974-025-03356-z.

## Introduction

Shiga toxin (Stx)-producing enterohemorrhagic Escherichia coli (EHEC) are responsible for significant epidemics globally, often resulting in severe organ damage and mortality rates [1]. Approximately 15% of EHEC-infected individuals develop the clinical triad of thrombocytopenia, haemolytic anaemia, and renal injury, collectively known as haemolytic uremic syndrome (HUS) [2].

Of concern, central nervous system (CNS) complications manifest in 30% of HUS cases [3]. In addition to the pathogenicity attributed to Stx, lipopolysaccharide (LPS) serves as a critical virulence factor in EHEC infections, exacerbating the deleterious effects of Stx2 and contributing to organ damage in the brain [4–6]. Stx-derived encephalopathy has been associated with alterations in memory and consciousness, seizures, and coma, highlighting the severity of CNS involvement in EHEC infections [7]. A multicentre, observational, retrospective, and cross-sectional study conducted by the National Epidemiological Surveillance System of Argentina concluded that central nervous system (CNS) involvement by Shiga toxin-producing *E. coli* (STEC) was the main predictor of death in patients with HUS [8].

Neuroinflammation plays a pivotal role in the neurodegenerative processes observed during STEC infection, prompting the exploration of anti-inflammatory interventions as potential therapeutic strategies [9, 10]. Microglia, as resident immune cells of the central nervous system, play essential roles in maintaining tissue homeostasis and orchestrating inflammatory responses [11–15]. However, their response to EHEC infections and their contribution to brain inflammation and injury remain incompletely understood [16–18]. Stx has been implicated in the activation of microglial cells, leading to increased metabolic activity, phagocytic capacity, and a pro-inflammatory phenotype [19]. Moreover, microglial activation has been implicated in injury and inflammation across various CNS pathologies, suggesting a potential role for these cells in the pathophysiology of STEC-HUS [20]. Among the inflammatory molecules implicated in EHEC infections, tumour necrosis factor alpha (TNF-α) has emerged as a key mediator. TNF-α activates macrophages in the kidney during STEC-HUS and can potentiate the effects of Stx on human endothelial cells [9, 21, 22]. Additionally, microglia have been shown to secrete TNF-α in various disease contexts, suggesting a potential crosstalk between endothelial cells and microglia [23]. Herein, we aimed to investigate the influence of TNF-α on the brain vasculature and the myeloid compartment in STEC-HUS. Leveraging state-of-the-art proteomics and a novel scalable machine learning algorithm for the segmentation of phenotypically complex microglial cells, we sought to elucidate TNF-α-dependent molecular and cellular changes in the brain, with the goal of identifying potential therapeutic targets implicated in brain inflammation and injury in STEC-HUS.

## Materials and methods

### Animals

Mice were bred and housed under specific pathogen-free conditions at the central animal facility at the University Hospital Essen and were allowed to eat and drink *ad* libitum. Male C57BL/6 mice (WT), 8–12 week-old mice from Charles River were used and individual mice were analyzed throughout the study. The following solutions were intravenously injected into the experimental groups: (i) Control (Ctrl) mice: 100 µL PBS as control solvent, (ii) Shiga toxin 2 (Stx, 0.4 ng/mouse in 100 µL, Toxin Technologies) in combination with LPS, 5.625 µg/mouse in 100 µL), (iii) Etanercept (0.2 mg/mouse in 100 µL PBS, (Enbrel) Pfizer) was applied 30 min after injection Stx/LPS. To evaluate whether the clinical aspects of an EHEC infection are recapitulated, erythrocyte fragmentation, circulation of free hemoglobin as an indicator for hemolytic anemia, thrombocytopenia and the increase of kidney injury markers creatinine and BUN in the serum of STEC-HUS mice were determined. At the indicated timepoints, mice were sacrificed via exposure to gradually increasing concentrations of isoflurane and cervical dislocation by the same operator.

### Immunofluorescence microscopy

Brains were collected 24 h after Stx/LPS injection, washed with PBS and fixed with 0.05 M phosphate buffer containing 0.1 M L-lysine (pH 7.4, Roth), 2 mg/mL NaIO4 (Roth), and 10 mg/mL paraformaldehyde (Sigma-Aldrich) overnight at 4 °C. Brains were equilibrated in 30% sucrose (Roth) solution for 24 h. Tissues were then frozen in OCT (Weckert Labortechnik) and stored at − 80 °C. Coronal Sects. (8 μm) of Bregma 0.445 mm were mounted on Super Frost Plus glass slides (R.Langenbrinck), dried for 10 min at 70 °C, rehydrated with PBS with 0.05% Triton X-100 (Roth) and blocked for 1 h with PBS containing 1% bovine serum albumin (GE Healthcare) and 0.05% Triton X-100. The staining was performed in blocking buffer (200μL per section). The TMEM119 (Abcam, Rabbit Recombinant Monoclonal TMEM119 antibody, ab209064, 1:100) followed by Anti-Rabbit 488 (Abcam, ab150061, Alexa488, 1:500), and CD64 (BioLegend, Biotin anti-mouse CD64 (FcγRI) Antibody, 139318, 1:100) antibody followed by Streptavidin 647 (Invitrogen (Molecular probes), S21374, Alexa647, 1:200) were each incubated for 1 h. Nuclei were stained with DAPI (2 mg/mL, Life Technologies, diluted 1:5000) for 5 min. Sections were imaged using the Zeiss Axio Observer.Z1 and Apotome (Zeiss) at the Imaging Center Essen, and the images were analyzed using the ZEN Software (Zeiss) and ImageJ.

### Protein extraction and tryptic digestion

Samples were diluted in 150 μL of 0.1 M triethyl ammonium bicarbonate (TEAB) and 1% (w/v) sodium deoxycholate SDC (SDC-buffer) and heated to 95 °C for 1 h. Sonification was performed for 6 pulses at 30% power. The protein concentration of denatured proteins was determined by the Pierce ™ BCA Protein assay kit following the manufacturer’s instructions. 20 μg of protein for each sample was diluted in 50 μL SDC-Buffer. Disulfide bonds were reduced with 10 mM DTT at 60 °C for 30 min. Cysteine residues were alkylated with 20 mM iodoacetamide (IAA) for 30 min at 37 °C in the dark. Tryptic digestion was performed for 16 h at 37 °C, using a trypsin (sequencing grade, Promega) / protein ratio of 1:100. After tryptic digestion the inhibition of trypsin activity as well as the precipitation of SDC was achieved by the addition of 1% formic acid (FA). Samples were centrifuged for 5 min at 16,000 *g*. The supernatant was dried in a SpeedVacTM vacuum concentrator and stored at − 20 °C until further use. Prior to mass spectrometric analysis, peptides were resuspended in 0.1% FA to a final concentration of 1 µg/µL. 1 µg was used for LC–MS/MS acquisition.

### High pH fractionation

13 high pH reversed phase chromatography fractions were used for spectral library generation. For fractionation equal amounts of all analyzed samples were dissolved in 10 mM NH_4_HCO_3_ to a final concentration of 1 µg/µL. In total 50 µg of digested proteins were used for High pH RP-HPLC using a 25 cm ProSwift™ RP-4H capillary monolithic column (Thermo Fisher) on an Agilent 1200 series HPLC (high pressure liquid chromatography) system. A gradient was applied for a total of 45 min with a flow rate of 0.2 mL/min starting at 96.7% eluent A (10 mM NH_4_HCO_3_) and 3.3% eluent B (10 mM NH_4_HCO_3_ in 90% acetonitrile (ACN)) for 5 min, rising to 38.5% B in 20 min increasing to 95.0% in 1 min for 10 min and re-equilibrated to 3.3% B for 8 min. 30 fractions were collected on an Äkta prime plus fraction collector, pooled to 13 fractions and dried in the vacuum centrifuge.

### LC–MS/MS data acquisition

Liquid-chromatography-coupled to tandem mass spectrometer (LC–MS/MS) measurements were performed using 20 µm thick coronal sections of Bregma 0.445 mm on a quadrupole-ion-trap-orbitrap MS (Orbitrap Fusion, Thermo Fisher) coupled to a nano-UPLC (Dionex Ultimate 3000 UPLC system, Thermo Fisher). Chromatographic separation of peptides was achieved with a two-buffer system (buffer A: 0.1% FA in water, buffer B: 0.1% FA in ACN). Attached to the UPLC was a peptide trap (180 µm × 20 mm, 100 Å pore size, 5 µm particle size, Symmetry C18, Waters) for online desalting and purification followed by a 25-cm C18 reversed-phase column (75 µm × 250 mm, 130 Å pore size, 1.7 µm particle size, Peptide BEH C18, Waters). Peptides were separated using an 80-min method with linearly increasing ACN concentration from 2 to 30% ACN in 60 min. Eluting peptides were ionized using a nano-electrospray ionization source (nano-ESI) with a spray voltage of 1800, transferred into the MS and analyzed in data dependent acquisition (DDA) mode. For each MS1 scan, ions were accumulated for a maximum of 120 ms or until a charge density of 2 × 10^5^ ions (AGC Target) was reached. Fourier-transformation based mass analysis of the data from the orbitrap mass analyzer was performed covering a mass range of 400–1300 m/z with a resolution of 120,000 at m/z = 200. Peptides with charge states between 2 + and 5 + above an intensity threshold of 1000 were isolated within a 1.6 m/z isolation window in Top Speed mode for 3 s from each precursor scan and fragmented with a normalized collision energy of 30% using higher energy collisional dissociation (HCD). MS2 scanning was performed, using an ion trap mass analyzer at a rapid scan rate, covering a mass range starting at m/z 120 and accumulated for 60 ms or to an AGC target of 1 × 10^5^. Already fragmented peptides were excluded for 30 s.

### Raw data processing

LC–MS/MS were searched with the Sequest algorithm integrated in the Proteome Discoverer software (v 2.41.15), Thermo Fisher Scientific) against a reviewed a mouse Swissprot database obtained in October 2020, containing 17,053 entries. Carbamidomethylation was set as fixed modification for cysteine residues and the oxidation of methionine, and pyro-glutamate formation at glutamine residues at the peptide N-terminus, as well as acetylation of the protein N-terminus were allowed as variable modifications. A maximum number of 2 missing tryptic cleavages was set. Peptides between 6 and 144 amino acids where considered. A strict cutoff (FDR < 0.01) was set for Peptide and protein identification. Quantification was performed using the Minora Algorithm, implemented in Proteome Discoverer. LC–MS/MS from High pH fractions were searched like the samples by adding the 13 fractions as a single file set in the Proteome Discoverer software. Afterwards, both result files were used to create a multi-consensus workflow and enhancing peptide identification including the match between runs function (MBR). Results were filtered for high confident peptides, with enhanced peptide and protein annotations. Protein abundances for individual samples were exported for subsequent statistical analysis. The data are available in accordance to FAIR principles (Supplementary data 1).

### Methods for microscopy image segmentation

A key requirement for reliable statistical analysis is to accurately segment nuclei, macrophages, microglia from microscopy images, from the DAPI, TMEM119, and CD64 channels, respectively. For nuclei segmentation from the DAPI channel, we trained a Cellpose model [24] with 10 manually annotated patches of roughly 300 × 300 pixels. For microglia and macrophage segmentation from the TMEM119 and CD64 channels, we built a deep neural network-based segmentation model with a human-in-the-loop strategy based on the iterative deep learning workflow concept presented in [25]. The workflow contained two main stages: the initialization stage (preparation of initial training set) and the iterative development stage (refining and enlarging the training set). First, we applied a top-hat filter to reduce the uneven illumination artefacts in the microscopy images as pre-processing. Then, during the initialization stage, an Otsu thresholding was applied on the pre-processed images to obtain preliminary segmentations, followed by manually cropping 14 representative images patches of roughly 500 × 500 pixels and manually fixing the inaccurate segmentation in these patches. We trained a HighResNet model [26] with these 14 manually annotated patches as the initial segmentation model, which yielded better segmentation than Otsu thresholding but still error prone. During the iterative development stage, we applied the latest segmentation model (either the initial model or the model from the previous iterative development cycle) on all full images and cropped additional representative error-prone patches into the training set after fixing the errors, to enlarge the training set. Then, we fine-tuned the model with the augmented training set. In total, we conducted two iterative development cycles until satisfactory segmentation can be obtained. Five and seven patches were added in the two iterative development cycles, respectively. At the end, we deployed the final segmentation model on all CD64 and TMEM119 images followed by applying a size filter to remove segmented pieces smaller than 45 pixels (14.62 µm) to generate the final cellular segmentation.

### Automated scalable image processing pipeline for data analysis

After developing the deep learning-based cellular segmentation models, a fully automated image processing pipeline was assembled using Snakemake for deployment [27] which enables easy-to-use, parallelised, and reproducible workflows that support a modular structure and are thus effortlessly extensible. Each module is defined by a rule that specifies inputs, outputs, python-scripts and additional requirements for each of the analysis steps in the Snakemake workflow. A YAML configuration file is provided with default options that can be customized at multiple levels, including the use of new machine learning models provided by the user. The workflow can be executed with a single console command. The input files should be in TIFF format with minimum meta data, including channel names and physical pixel sizes. CD64 and TMEM119 are declared in the configuration file as “target”. Since DAPI is segmented differently (Cellpose model) and used solely for registering, it is assigned separately in the configuration file as “registration”. This assignment makes the pipeline reusable for different datasets, as the channel names assigned to “target” and “registration” can be adjusted to fit the input images. The channel “registration” is first segmented using the previously described Cellpose model and dilated using skimage (v.0.22.0) [28]. The channels in “target” are first contrast enhanced followed by the application of the white top-hat filter (skimage). The resulting images are then segmented using the ML models, or optionally through the otsu-threshold (skimage), and subsequently post-processed to remove large objects, small objects, and small holes, which can be adjusted or turned off in the configuration file. After segmentation, each marker in “target” is individually registered using the marker declared in “registration” assigning each object in the dilated “registration” image to one object with the highest overlap from the “target” image. If an object from the “target” image overlaps more than 1 “registration” marker it is assigned to the one with the stronger pixel-based overlap. The registered objects from the “target” image are then skeletonized using Lee’s algorithm (skimage) with subsequent pruning of branches with length 1. The cell area analysis is conducted on the registered images and the structure analysis is performed on the skeletonized “target” images. The cell area in µm is computed as the number of pixels of an object in the registered “target” image multiplied by the physical pixel sizes. For the analysis of the longest path and the number of branches, endpoints and branchpoints are computed using graphs generated with networkx (v.3.2.1) and the skeletonized objects [29].

### Methods for macroscopy image analysis

Images of the brains were captured using the wide-angle camera of an Apple iPhone 12 mini, maintaining a consistent distance between the iPhone and the brains to ensure uniformity and prevent variations in brain dimensions from influencing the analysis. A color threshold was applied using Image J (Hue Min 0 Max 9; Saturation Min 114 Max 255; Brightness Min 0 Max 241) resulting in vascular binary masks. The total brain area was annotated by the same operator with the Image J select tool. The level of angiopathy was determined by calculating the blood vessel positive area (pixel) per brain area (pixel) as $Angiopathy \left[\%\right]= \frac{Area Blood Vessel \left(Pixel\right)}{Area Brain \left(Pixel\right)}.$ Kruskal Wallis with Dunn´s post hoc test was used to calculate the significance.

### Bioinformatics and statistical analysis

LC–MS/MS data was first normalized using loess normalization (R-limma) [30] followed by imputation using a uniform random 1st–5th percentile strategy for missing values of proteins in groups with less than 2 non-missing values, assuming they are missing not at random, and KNNImputer [31] for the remaining missing values. Effect sizes were estimated using log_2_ fold change (log_2_FC) and signal-to-noise ratio (SNR) where the *log*_*2*_FC is computed as ${log}_{2}\text{FC}={\text{log}}_{2}\left(\frac{\overline{{\text{x} }_{1}}}{\overline{{\text{x} }_{2}}}\right)$, with $\overline{{\text{x} }_{1}}$ and $\overline{{\text{x} }_{2}}$ as the mean values of the conditions, and the SNR is calculated using $SNR=\left(\frac{\overline{{x }_{1}}-\overline{{x }_{2}}}{{d}_{1}+{d}_{2}}\right)$ with d1 and d2 representing the respective standard deviation. Statistical significance was assessed using the Welch’s t-test. Significant regulation was considered for the proteins with Log_2_FC > ± 1 and P-value < 0.05. Heatmap, violin, box, bar, and volcano plots and principal component analysis (PCA) were generated using the Python package matplotlib (v.3.8.1) [32] and seaborn (v.0.13.2) [32, 33]. Clustermap (seaborn) with hierarchical clustering was computed using protein-wise MinMaxScaled (scikit-learn) values and the farthest neighbor clustering algorithm (scipy-linkage) [34] on the computed pairwise euclidean distance matrix (scipy-pdist) for proteins and default options for sample clustering. Protein subclusters were determined using a threshold of 0.68 times the maximum of all distances from the distance matrix. Subcluster significance was assessed using correlation between Stx vs Stx + Eta&Control.

### Data availability

Processed mass spectrometry data are available as supplemental tables. The mass spectrometry raw data have been deposited to the ProteomeXchange Consortium via the PRIDE partner repository with the dataset identifier PXD052925. For the peer review process data are available through the following login details: Username: reviewer_pxd052925@ebi.ac.uk; Password: Mf7TI6a1byrA. The code for the image analysis pipeline is openly available on GitHub https://github.com/MMV-Lab/immun_analysis. STRING analysis were conducted with significantly regulated proteins and the most significant subcluster [35].

Key resource table

**Table:** 

Reagent or resource	Source	Identifier
Antibodies and antibody binding agents		
Abcam, Rabbit Recombinant Monoclonal 105 TMEM119 antibody, ab209064	Abcam Cat# ab209064	RRID:AB_2800343
Donkey Anti-Rabbit IgG H&L (Alexa Fluor^®^ 488) preadsorbed	Abcam Cat# ab150061	RRID:AB_2571722
Biotin anti-mouse CD64 (FcγRI)	BioLegend Cat# 139318	RRID:AB_2566557
Streptavidin, Alexa Fluor^®^ 647 conjugate	Thermo Fisher Scientific Cat# S-21374	Cat# S-21374
Reagents and Instruments		
Shiga toxin	Toxin Technologies	STX2-BB12
Etanercept (Enbrel ^**®**^)	Pfizer	–
Bovine serum albumin	GE Healthcare	Cat #K45-001
DAPI	Life Technologies	Cat #D1306
Triethyl ammonium bicarbonate (TEAB)	Thermo	Cat #90114
Sodium deoxycholate (SDC)	Thermo	Cat #89904
Pierce BCA Protein Assay Kit	Thermo Fisher	RRID: SCR_008452
Dithiothreitol (DTT)	Roche	Cat #10708984001
Iodoacetamide (IAA)	Sigma	Cat #I1149
Trypsin, sequencing grade	Promega	
Formic acid (FA)	Biosolve	Cat #00069141
SpeedVac™ vacuum concentrator	Thermo Fisher	–
ProSwift™ RP-4H capillary monolithic column	Thermo Fisher	–
Agilent 1200 series HPLC system	Agilent Technologies	–
Orbitrap Fusion mass spectrometer	Thermo Fisher	RRID: SCR_020562
Dionex Ultimate 3000 UPLC system	Thermo Fisher	–
Peptide trap (Symmetry C18)	Waters	–
Peptide BEH C18 column	Waters	–
L-lysine	Sigma-Aldrich	Cat #L5626
Paraformaldehyde	Sigma-Aldrich	Cat #P6148
PBS	Life Technologies	Cat #18912-014
Sucrose	Carl Roth GmbH	Cat #9097.1
Tissue-Tek OCT	Sakura	Cat #4583
Triton X-100	Carl Roth GmbH	Cat #3051.4
Experimental Models: Mouse Strains		
C57BL/6J	Jackson Laboratories	RRID:IMSR_JAX:000664
Software		
Adobe Illustrator CC 2018–2019	Adobe	RRID:SCR_010279
Fiji	ImageJ	RRID:SCR_003070
FlowJo 10	FlowJo	RRID:SCR_008520
GraphPad Prism, version 10	GraphPad Software	RRID:SCR_002798
R Project for Statistical Computing, version 3.5.1	https://cran.r-project.org/	RRID:SCR_001905
ZEN Digital Imaging for Light Microscopy, ZEN 2012	Zeiss	RRID:SCR_013672

## Results

**Fig. 1:**
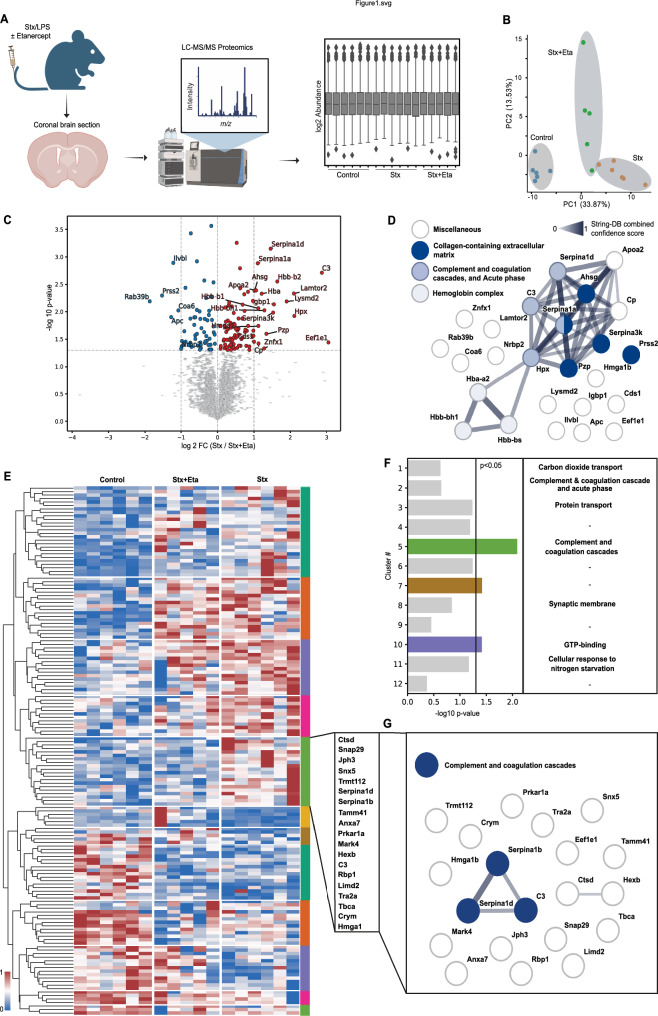
Inhibition of TNF-α reduces the activation of the complement and coagulation cascade. **A** Schematic illustration of the measurement of coronal brain section of mice by liquid chromatography mass spectrometry (LC–MS/MS). Generated by Biorender. **B** Dimensionality reduction of the proteins with P < 0.05. **C** Statistical analysis (log2 fold change (log2FC) and P-value) of the proteins enriched in Stx/LPS-treated mice in comparison to TNF-α inhibition (Stx + Eta). **D** STRING network of the significantly regulated proteins between “Stx” and “Stx + Eta” (P < 0.05, fold change (Fc) < − 1 and > 1) indicated enrichment of specific pathways. **E** and **F** Hierarchical clustering of the significantly regulated proteins (P < 0.05) indicated 12 distinct clusters (**E**). Correlation analysis (“Ctrl” and “Stx + Eta” versus “Stx”) determined cluster 5 as the most differentially regulated (**F**). **G** STRING analysis of proteins of cluster 5. n = 5 (Stx and Eta + Stx) and 6 (Ctrl)

In a murine model of Enterohemorrhagic Escherichia coli (EHEC) infection, we administered a combination of Shiga toxin (Stx) and lipopolysaccharide (LPS) via intravenous injection into mice, followed by treatment with the TNF-α inhibitor Etanercept (Fig. 1A). Liquid chromatography tandem mass spectrometry (LC/MS/MS) on coronal brain sections coupled with a systems biology approach was used to elucidate TNF-α-dependent inflammatory proteomic alterations (Fig. 1A). Dimensionality reduction using principal component analysis (PCA) of significantly regulated proteins revealed substantial alterations in the brain proteome following Stx administration, as indicated by the strong separation of the ctrl versus Stx group in the component 1 (Fig. 1B). Along this principal component, which accounted for 33.87% of the variation, the group treated with Etanercept located as an intermediate population between the control and Stx groups, suggesting partial mitigation of the proteomic changes (Fig. 1B). In addition, this treatment group also dominated the second principal component, indicating unique proteomic changes induced by this treatment (Fig. 1B). Comparative analysis of proteins in Stx/LPS-treated mice with and without Etanercept treatment revealed significant upregulation of molecules such as C3 and Serpina1a/1d upon Stx and LPS injection, which were attenuated by TNF-α inhibition (Fig. 1C, Supplementary data 1).

Analysis of the significantly regulated proteins using STRING highlighted enrichment of pathways including “Complement and coagulation cascades,” “Acute phase,” “Haemoglobin complex,” and “Collagen-containing extracellular matrix” (Fig. 1D). Hierarchical clustering identified 13 main protein clusters across experimental groups, with cluster 5 showing the most pronounced regulation following TNF-α inhibition (Fig. 1E). Pathway analysis of proteins within cluster 5 reaffirmed “Complement and coagulation cascade” enrichment, suggesting that TNF-α inhibition mitigates angiopathy via reduced endothelial damage (Fig. 1G).

**Fig. 2:**
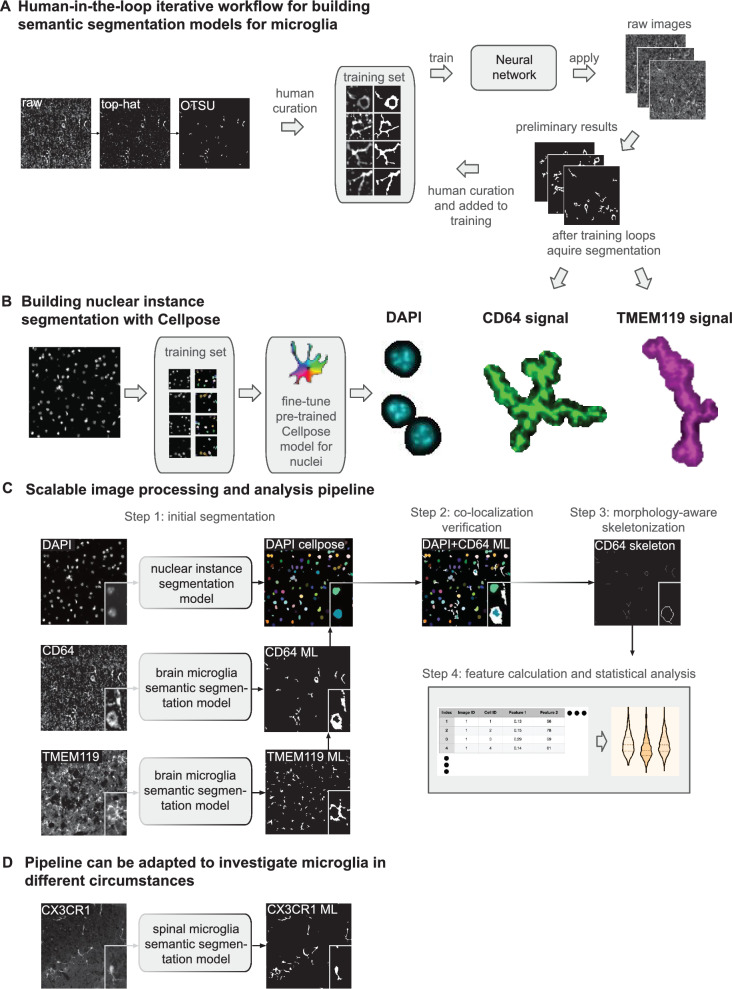
Scalable, open-source ML pipeline for the quantitative and qualitative analysis of microglia. **A** The highlighted cortical region of the brain of Bregma 0.445 mm (mouse.brain.map.org) was used for the human-in-the-loop iterative workflow, consisting of human curation after Top-Hat and OTSU thresholding. A neural network was trained and applied to raw images to segment CD64 and TMEM119 signals. **B** Nuclear instance segmentation with Cellpose to segment DAPI + nuclei. **C** Scalable image processing pipeline for feature calculation and statistical analysis of microglia. **D** Adaptation of ML pipeline to other microglia markers and tissues

Given the pivotal role of microglia in brain inflammation and regeneration, we investigated whether proteomic alterations were associated with phenotypic changes in the microglial populations. Since microglia have a complex morphological architecture with distinct expression of CD64 and TMEM119 for BM and Yolk-sac origin, we developed a human-in-the-loop iterative workflow for semantic segmentation modelling of microglia expression CD64 or TMEM119 (Fig. 2A). We first established semantic segmentation for CD64 and then adapted the workflow for TMEM119 (Fig. 2A). For the cell nucleus, we utilized the Cellpose algorithm for nuclear instance segmentation (Fig. 2B). Our scalable image processing pipeline facilitated analysis across experimental conditions (Figure S1 and Fig. 2C) and could also be applied to CX3CR1 signal in the central nervous system (Fig. 2D), demonstrating applicability beyond the brain.

**Fig. 3:**
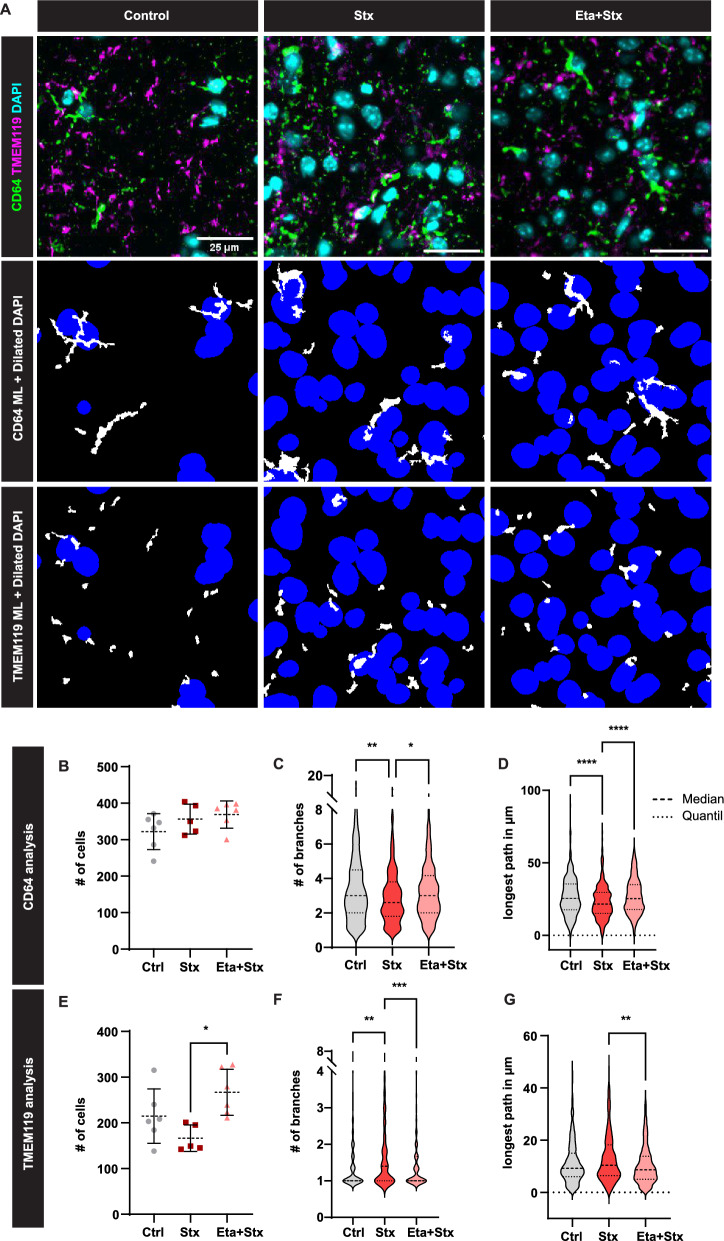
TNF-α inhibition prevents disease-mediated phenotypic changes of microglia. **A** Application of the ML pipeline to cortical brain regions as indicated in Fig. 2A to segment CD64 + (BM-derived) and TMEM119 + microglia (yolk sac-derived). **B–G** Quantitative and phenotypic analysis of microglia populations using the ML pipeline. Mean and SD are indicated in (**B, E**). Median and Quantil are indicated by the dashed lines (**C, D, F, G**). Kruskal Wallis with Dunn’s post hoc test, *P < 0.05, **P < 0.01, ***P < 0.001, ****P < 0.0001. Number of mice used: n = 5 (Stx and Eta + Stx) and 6 (Ctrl) (B to G); Bar in (**A**) indicates 25 µm

Analysis of microglial populations indicated unaltered numbers (Fig. 3A,B) but diametric phenotypic changes following Stx/LPS injection (Fig. 3C,D). Specifically, CD64 + microglia exhibited reduced branching, while TMEM119 + cells showed increased branching upon Stx/LPS injection. Both changes were restored to baseline levels after TNFα inhibition (Fig. 3C,D). Our machine learning-based segmentation algorithm underscored significant phenotypic alterations in microglia post-Stx/LPS administration and highlighted TNF-α inhibition-associated changes, potentially contributing to reduced angiopathy.

**Fig. 4:**
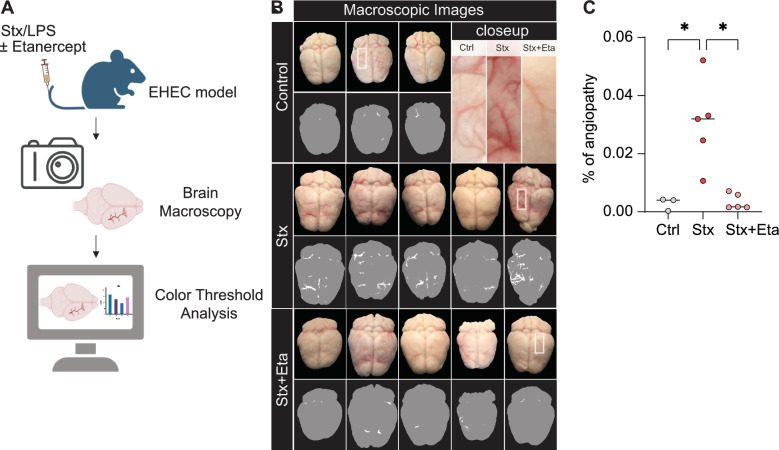
Reduced brain angiopathy after inhibition of TNF-α. **A** Schematic overview of the experimental design. Generated by Biorender. **B** Macroscopy of the brain tissue indicated phenotypic changes of blood vessels after inhibition of TNF-α through Etanercept (Stx + Eta). A color threshold was applied using Image J (Hue Min 0 Max 9; Saturation Min 114 Max 255; Brightness Min 0 Max 241) to all images resulting in vascular binary masks to quantify angiopathy in the brain macroscopies. White refers to the blood vessels and grey indicates the brain tissue. **C** Quantification of the vascular angiopathy upon injection of shiga toxin (Stx) and etanercept (Stx + Eta). The level of angiopathy (%) was determined by calculating the blood vessel positive area (pixel) per brain area (pixel). Kruskal Wallis with Dunn’s post hoc test, bar represents the mean, *P < 0.05. n = 5 (Stx and Eta + Stx) and 3 (Ctrl)

To analyze brain injury and vasodilation, a macroscopic examination and image analysis was used (Fig. 4A). We observed pronounced angiopathy in mice exposed to Stx and LPS compared to untreated controls (Fig. 4B). Inhibition of TNF-α with Etanercept significantly attenuated brain angiopathy (Fig. 4B). Quantitative image analysis confirmed significant angiopathy in the brains of Stx/LPS-treated mice, with mitigation of vascular pathology following TNF-α inhibition (Fig. 4C). Thus, macroscopic imaging underscored the crucial role of TNF-α in angiopathy induced in the murine EHEC model.

## Discussion

Infections with enterohemorrhagic *E. coli* (EHEC) result in significant multi-organ consequences. Kidney damage, leading to haemolytic uremic syndrome (HUS), as a prominent manifestation [9]. Despite the severity of the disease, a specific therapy for EHEC-induced HUS remains elusive. Antibiotic treatments have been shown to exacerbate the release of Shiga toxins due to bacterial stress. Inhibition of the complement cascade was attempted during the major 2012 outbreak in Germany yet failed to yield significant improvements in disease progression [36–38]. Expanding upon our previous findings, the present study extends the critical examination of disease symptoms to include the brain. Our study shows a clear macroscopic change of the vasculature in the brain after anti TNF-α therapy. Furthermore, we show significant molecular changes and identify proteins that may contribute to the macroscopic changes of the blood vessels. Microscopy revealed a significant phenotypic change in microglia and identified proteins that lead to the reduced activation during anti TNF-α therapy using deconvolution algorithms. Our study sheds light on the intricate mechanisms underlying the neuroinflammatory response in EHEC infection, particularly focusing on the role of TNF-α in brain pathology. Through a comprehensive approach integrating in vivo experiments, proteomic analysis, and advanced ML-guided microscopy techniques, we elucidated TNF-α-dependent molecular and cellular changes, providing valuable insights into potential therapeutic targets for mitigating neuroinflammation and injury associated with EHEC infection.

Anti-inflammatory agents have emerged as important modulators in STEC-HUS [39]. Among others, the drug etanercept, which blocks the inflammatory molecule TNF-α, has been reported to protect animals against a lethal dose of Stx2 by reducing neuronal Stx2 uptake [40]. Notably, significant overexpression of TNF-α transcripts in the brain has been observed following Stx2 challenge [10]. As etanercept promotes the clearance of TNF-α, it may prevent Gb3 neuronal up-regulation induced by TNF-α and other pro-inflammatory cytokines [40, 41]. Moreover, the details of the inflammatory and stress responses leading to brain injury are crucial for an understanding of Stx-mediated neuropathogenesis. Brain endothelial cells are essential parts of the blood–brain barrier (BBB), maintaining brain function by blocking the passage of macromolecules [42]. Our data indicate a strong and TNF-α-dependent vascular injury in EHEC infections. The role of non-vascular cell types in the neuropathogenesis of Stx-mediated disease is constantly emerging. Astrocytes produce TNF-α when stimulated with Stx and LPS, increasing vascular permeability and susceptibility of endothelial cells to Stx through elevated Gb3 expression [43]. Treatment of Stx/LPS-stimulated astrocytes with the TNF-α inhibitor Etanercept, ameliorated these pathological events [43]. Our data indicate that therapeutic inhibition of TNF-α via Etanercept significantly reduced the vascular changes in the brain suggesting a TNF-α-dependent inflammatory crosstalk between non-vascular and endothelial cells leading to brain injury upon Stx challenge. Moreover, damaged endothelial cells may release pro-inflammatory cytokines, such as TNF-α, which upregulate the production of acute-phase proteins (e.g., Serpina1a/1d) and complement factors (e.g., C3) as part of the vascular inflammatory response, as seen by the LC–MS/MS analysis. By dampening this effect, Etanercept could reduce endothelial damage, limiting complement activation (C3 levels) and subsequent tissue injury. Blocking TNF-α might also decrease innate leukocyte activation, such as neutrophils, lowering the need for mitigating neutrophil elastase and other protease activities by Serpina1a/1d.

The myeloid compartment in the brain plays a critical role in maintaining homeostasis. Microglial cells represent an integrated myeloid population with distinct phenotypes, origins, and functions [15, 44, 45]. Previous studies have suggested a fundamental role for microglia in the pro-inflammatory process following Stx2 intoxication [19]. Responses to neural injury include up-regulation of surface-marker expression (e.g., Iba1) and stereotypical morphological changes [46]. To study the morphological changes in EHEC infections, we used our machine learning-based image analysis, which revealed an altered phenotype of CD64 + and TMEM119 + microglia, with these changes being restored after TNF-α inhibition, indicating reduced microglial activation in the absence of TNF-α.

Limitations of the study: This study used a mouse model to mimic EHEC infection in humans. Although this preclinical model has widely been used, the mechanisms in mice might not fully replicate the complexity of the human EHEC infection and its progression. In addition to TNF-α, other inflammatory cytokines (e.g., IL-1β, IL-6) might contribute to the observed brain pathology and may act in concert in the human situation. The inflammatory response, and in particular the inhibition of TNF-α, was performed systemically, preventing conclusions on whether systemic or local mechanisms in the brain are responsible for the observed findings. Furthermore, untargeted LC–MS/MS proteomics has identified numerous protein classes and pathways but may miss low-abundance proteins or post-translational modifications critical to the disease mechanism. Finally, the novel human-in-the-loop deep learning algorithm might introduce bias during training of results. The robustness of these findings needs further validation across different datasets or imaging platforms.

Overall, our study provides compelling evidence for the critical role of TNF-α in EHEC-induced brain pathology and highlights potential therapeutic targets for mitigating neuroinflammation and injury. We describe a human-in-the-loop deep learning-based segmentation framework, which is easier to use in practice comparing to common practices (e.g., manually labeling many images as ground truth and then train a neural network for segmentation), and easily adaptable to other segmentation problems. Using this microscopy framework and state-of-the-art MS, our findings underscore the importance of targeting TNF-α-mediated pathways to alleviate cellular and molecular alterations, such as vascular damage, proteomic alterations, and microglial activation associated with EHEC infection. Future therapeutic strategies aimed at modulating TNF-α signalling hold promise for improving outcomes in patients with EHEC-induced neurological complications, including haemolytic uremic syndrome (HUS) and central nervous system (CNS) involvement.

## Supplementary Information

Additional file 1.

Additional file 2.

## Data Availability

The mass spectrometry raw data have been deposited to the ProteomeXchange Consortium via the PRIDE partner repository with the dataset identifier PXD052925. For the peer review process data are available through the following login details: Username: reviewer_pxd052925@ebi.ac.uk; Password: Mf7TI6a1byrA. The code for the image analysis pipeline is openly available on GitHub https://github.com/MMV-Lab/immun_analysis.
